# Local edge-enhanced active contour for accurate skin lesion border detection

**DOI:** 10.1186/s12859-019-2625-8

**Published:** 2019-03-14

**Authors:** Mustafa Bayraktar, Sinan Kockara, Tansel Halic, Mutlu Mete, Henry K. Wong, Kamran Iqbal

**Affiliations:** 1 Bioinformatics, University of Arkansas Little Rock, 2804 S. University, Little Rock, 72204 AR USA; 2 Computer Science, University of Central Arkansas, 201 Donaghey Avenue, Conway, 72035 AR USA; 3 Computer Science, Texas A&M University-Commerce, 2200 Campbell, Commerce, 75428 TX USA; 4 Dermatology, University of Arkansas for Medical Sciences, 324 Campus Dr., Little Rock, 72205 AR USA; 5 Systems Engineering, University of Arkansas for Medical Sciences, 2804 S. University, Little Rock, 72204 AR USA

**Keywords:** Dermoscopy, Skin lesion segmentation, Skin lesion border detection

## Abstract

**Background:**

Dermoscopy is one of the common and effective imaging techniques in diagnosis of skin cancer, especially for pigmented lesions. Accurate skin lesion border detection is the key to extract important dermoscopic features of the skin lesion. In current clinical settings, border delineation is performed manually by dermatologists. Operator based assessments lead to intra- and inter-observer variations due to its subjective nature. Moreover it is a tedious process. Because of aforementioned hurdles, the automation of lesion boundary detection in dermoscopic images is necessary. In this study, we address this problem by developing a novel skin lesion border detection method with a robust edge indicator function, which is based on a meshless method.

**Result:**

Our results are compared with the other image segmentation methods. Our skin lesion border detection algorithm outperforms other state-of-the-art methods. Based on dermatologist drawn ground truth skin lesion borders, the results indicate that our method generates reasonable boundaries than other prominent methods having Dice score of 0.886 ±0.094 and Jaccard score of 0.807 ±0.133.

**Conclusion:**

We prove that smoothed particle hydrodynamic (SPH) kernels can be used as edge features in active contours segmentation and probability map can be employed to avoid the evolving contour from leaking into the object of interest.

## Background

Image segmentation is a process of finding meaningful regions in an image. Many of the image processing and analysis methods rely on the accuracy of a proper image segmentation method. In dermoscopic image processing and analysis, image segmentation corresponds to detection of lesion border precisely. Accuracy of skin lesion border detection in dermoscopic images is critical [1] to extract important structural features, such as irregularity, symmetry, and abrupt border cutoff; and dermoscopic features, such as globules, blue-white areas, and atypical pigment network. However, automated border detection is a challenging task especially among the lesions with a) fuzzy borders, b) low contrast between lesion boundary and surrounding skin, c) low color and texture variations, and d) existence of artifacts such as sweat, hair, and blood vessels.

In the USA approximately 3.5 million people are diagnosed with skin cancers in a year. Skin cancer is rarely fatal except for melanoma, which is malignancy of melanocytes [2]. In its January 2017 report, American Cancer Society estimates that in the U.S. 87,110 adults will be diagnosed with melanoma, and approximately 9730 cases are expected to be fatal [2]. Since melanoma develops in melanocytes, which are special cells on epidermis, it can be detected by visual inspection of skin. Early diagnosis and treatment of melanoma are key to increase chances of survival [3]. However, high rate of false-negative diagnosis in melanoma cases poses challenge for early treatments [3].

Dermoscopy is an effective and noninvasive imaging modality in diagnosis of skin cancers, especially for pigmented lesions. It enables clinicians to closely examine predefined diagnostic features that are not seen otherwise. For this very reason, accurate skin lesion border detection is key to extract important dermoscopic features of the lesion. These features are evaluated to detect melanoma and other skin diseases [4–7]. It is shown that dermoscopy increases accuracy of naked eye examination of clinicians [8]. There are various methods used to segment skin lesions [9]. One of these methods is using the algorithm of active contour.

Active contour based methods (a.k.a. snakes) are widely used in image segmentation. These methods are also used in lesion segmentation [10–13]. Active contours can be categorized into two main groups: edge-based methods [14] and region-based methods [15]. The former employs edge information [14] while the latter selects a region feature to adjust the movement of active contour toward the boundary of object(s) to be segmented [16, 17]. Active contour methods start with a curve around the region of interest (ROI) to be detected, the curve moves toward its interior normals and has to stop on the boundary of the ROI. While some parameters control the smoothness of the contour, others attract the contour toward the center of the ROI. The most optimum state of the contour is selected using an iterative process, in which internal and external energy functions reach equilibrium and stop the further iterations. Edge based active contours use level sets and have the advantage of handling complicated shapes. However, their parameters are not naturally connected to visual features; therefore, very difficult to use for naive users. The edge based active contours are found more suitable for lesion boundary detection [10, 11]. On the other hand, for border detection of skin lesions, active contours were reported [13] to have slower computation time since they require to solve the underlying optimization problem. In general, for an active contour method to achieve high accuracy for skin lesion detection, the lesion is expected to have strong edges to stop at the border.

Edge-based active contour methods suffer from poorly defined edges, whereas region-based methods are sensitive to inhomogeneity of image intensities. For the images with weakly formed object boundaries (e.g., skin lesions with fuzzy borders), the edge-stop function (ESF) fails to cease the curve move and as a result contour leaks through the object border [18]. Thus, they suffer in skin lesion segmentation when morphological and color variations exist. Specifically, for the cases where skin lesion doesn’t have a strong border (e.g., fuzzy borders, or insufficient contrast between lesion boundary and surrounding skin), active contour methods fail to find lesion borders accurately. One of the main contributions of this study is to overcome this failing point. The proposed method of segmentation starts with a novel local edge extraction algorithm using smoothed particle hydrodynamics (SPH). Using the edge information coming from SPH, object border is strengthened using geodesic distances that involves probability of pixels (whether they are foreground, background, or border pixels). Later we give the object edge information into active contours to accurately detect skin lesion borders. Due to the additional edge information given to active contours, they become robust to leaks.

**Fig. 1:**
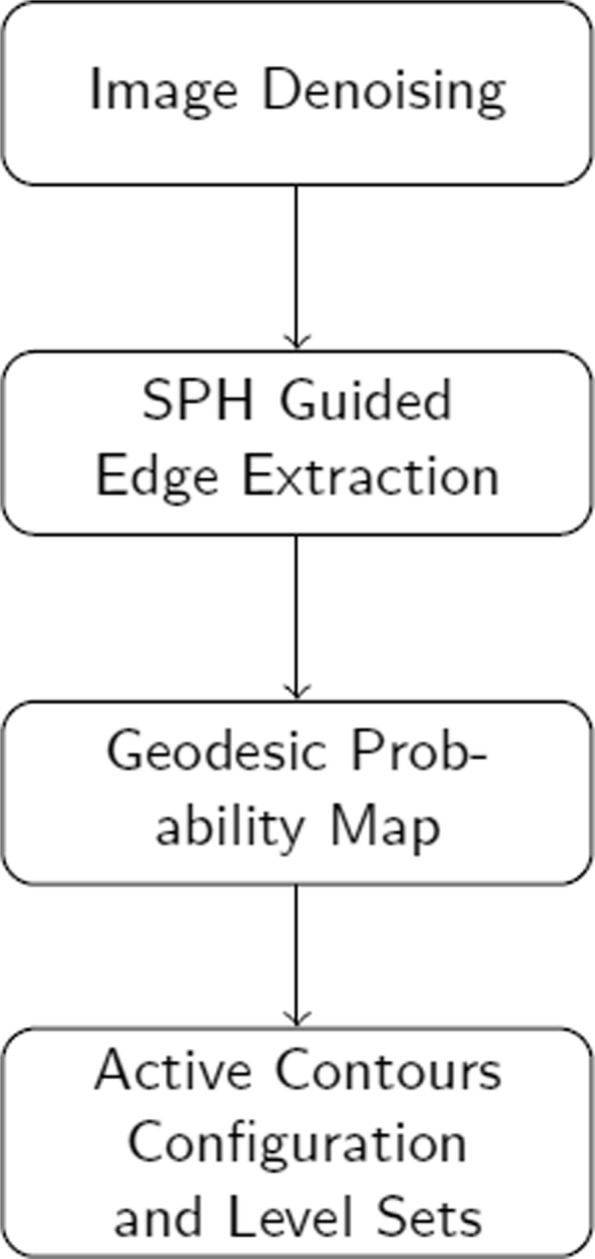
LEEAC takes the original dermoscopy image and generates segmented lesion as shown in the second row. It represents computational pipeline where LEEAC takes image creates background and foreground patches, denoises image, extracts local edge features with SPH, generates probability maps to further eliminate leaking problem, applies active contour, and finally generates the final segmented image using level sets

Process flow of skin lesion border detection with Local Edge-Enhanced Active Contour (LEEAC) is as follows (see Fig. 1). We first apply intermeans thresholding [19] on the given dermoscopy image. This leads us to coarsely locate lesion pixels and background pixels to extract sample patches which will be used for background/foreground probability map. Then we perform image filtering using Perona-Malik [20] denoising method to eliminate active contours trapped at relatively strong edges at the background pixels. Later, SPHs are calculated to find local edges of an image. We incorporate Probability Maps in to SPH kernels in order to make the lesions’ edges even stronger. In this study, it is proven that probability maps incorporated with SPH kernels are robust edge indicator functions that eliminate unwanted leakage problems [18] encountered in active contours. This novel SPH based robust edge indicator function is then solved using Level Sets [14], which in turn generates accurate skin lesion border detection even for lesions with fuzzy borders.

## Methods

This section reviews the developed computational core for lesion segmentation. Figure 1 shows its processing steps. Each of these steps are detailed in the following subsections.

### Filtering

We use Perona-Malik filtering [20] method that aims to smooth noises while preserving significant information, in our case edges. Perona-Malik filtering is chosen since it preserves edges. Formal representation of this filtering method is as follows;

1 $$  g(\nabla(I))=\frac{1}{1+\sqrt{1+\frac{{\nabla(I)}^{2}}{\gamma^{2}}}}  $$

where *g*∇(I) represents the diffusion coefficient, ∇(I) gradient map of the image *I*. As can be inferred from the Eq. 1, ∇(I) and g are inversely proportional to maintain the notion of Perona-Malik method. *γ* is a constant to control the sensitivity against gradients on the image domain. Diffusion process will be declined at the regions where ∣∇*I*∣≫*γ*. Without smoothing, initial contour is trapped by noise(s) (weak edges) and cannot delineate the lesion border. After denoising completed, SPH kernel is used to overcome active contour leaking problems, especially for the fuzzy borders of skin lesions.

### A new local edge extraction method: SPH

SPH is an interpolation method which is used for numerically solving diffusion equations. It is used in various applications such as highly deformable bodies simulations, lava flows, and computational fluid dynamics. Its principle is based on dividing the fluid (medium) into a set of discrete elements which are called particles. Equation 2 describes this interpolation where h is the length of smoothing function W, and r is the descriptor of the medium entity, r ^′^ is the adjacent entities in the range of h. There are many kernel functions defined in the literature [21, 22]. One of them is Monoghan’s cubic spline [21] as given in Eq. 2 that provides the temperature (in its specific case) A, at position r relying on the temperatures of all particles in a radial distance h.

2 $$  A_{t}(r)= \int \mathrm{A}(r^{\prime}) \mathrm W\{ |r-r^{\prime}|,h\} \mathrm{d}r^{\prime}  $$

In Eq. 2, the contribution of a particle to a physical property are weighted by their proximity to the particle of interest and its density. Commonly used kernel functions deploy cubic spline and Gaussian functions. Cubic spline is exactly zero for particles located at a distance equals two times of the smoothing length, 2h. This decreases the computational cost by discarding the particles’ minor contributions to the interpolation.

To expand the representation of SPH kernel in physics, let us take another particle *j* and associate it to a fixed volume *ΔV*_*j*_ with a lump shape, which leads determining the computational domain with a finite number of particles. Concerning the mass and density of the particle, the lump volume can be rewritten as the ratio of mass to density *m*_*j*_/*p*_*j*_ [23]. Mathematical representation is given in the following equation,

3 $$  \mathrm{A}(r)= \sum\limits_{j} m_{j} \frac{A_{j}}{p_{j}}W\left(\left| r-r_{j} \right|\right),h)  $$

where *A* is any quantity at r; *m*_*j*_ is the mass of particle *j*; *A*_*j*_ is the value of the quantity *A* for particle *j*; *p*_*j*_ is the density of particle *j*; *r* is spatial location; and W is the kernel function. The density of particle *i*, *p*_*i*_ can be expressed as in the Eq. 4.

4 $$ {\begin{aligned}  \rho_{i}= \mathrm{(\rho(r_{i}))}&=\sum\limits_{j} m_{j} \frac{p_{j}}{p_{j}}W\left(\left| r-r_{j} \right|\right),h)\\&= \sum\limits_{j} m_{j} W\left(\left| r-r_{j} \right|\right),h) \end{aligned}}  $$

where the summation over j covers all particles. Since *m* is a scalar, gradient of a quantity can be found easily by the derivative ∇ as seen in Eq. 5.

5 $$  \nabla \mathrm{A} (r)= \sum\limits_{j} m_{j} \nabla W\left(\left| r-r_{j} \right|\right),h)  $$

### Kernel approximation

A feasible kernel must have two following properties,

6 $$  \int\limits_{\Omega}\mathrm{W(r,h) }= 1  $$

and

7 $$  {\lim}_{h \to 0} \mathrm{W} (r,h)=\delta(r)  $$

where *δ* is the Dirac Delta function.

8 $$  \delta(r)= \left\{\begin{array}{ll} \infty, & \,\, \text{if}\ a=0 \\ 0, & \,\, \text{otherwise} \end{array}\right\}  $$

Kernel must be an even function and greater than zero all time [23]. These cases are expressed formally as in the following;

9 $$  \mathrm{W(r,h)} \geq 0 \hspace{1 cm}\text{and}\hspace{1 cm} \mathrm{W(r,h)}=\mathrm{W(-r,h)}  $$

Several kernels for SPH are proposed including Gaussian, B-Spline, and Q-spline [21, 22, 24]. Even though, Q-spline is considered the best in [24] in terms of accuracy, it is computationally expensive due to the square root computations. We propose to use 6^*th*^ degree polynomial kernel suggested by [24] as the default kernel, which is expressed below,

10 $$  W_{default}(r,h)=\frac{315}{64*\pi*h^{9}}{\left(h^{2}- \left| r \right|^{2}\right)^{2}}  $$

with the gradient,

11 $$  \nabla W_{default}(r,h)=\frac{945}{32*\pi*h^{9}}{\left(h^{2}- \left| r \right|^{2}\right)^{2}}  $$

**Fig. 2:**
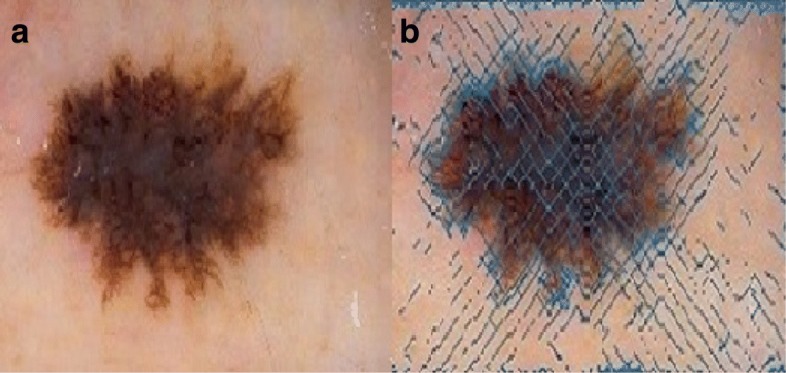
**a** A dermoscopy image; as can be seen in **b**, Blue lines represent normals of edges on the image, which later used for lesion border segmentation

Once SPH is applied to dermoscopy images, it generates all local edge features. Figure 2 illustrates edge features derived by SPH on a dermoscopy Image. In our experiments, we empirically selected 1 for h, and 6^*th*^ degree kernel for interpolation. Obtained SPH map will be used as the edge indicator function in the lesion border segmentation. Formal representation of the edge indicator function is given as in the Eq. 12,

12 $$  g=\frac{1}{1+\mid \nabla (G_{\alpha}*I) \mid^{p}}, p=1,2  $$

where *I* is the image, *G* is denoising function, and ∣∇(*G*_*α*_∗*I*)∣^*p*^ is the edge map produced by image gradients. In this paper, we used SPH formulations that are to calculate surface normals, instead of image gradients. Edge indicator functions are commonly represented by *g* as shown in Eq. 12. Next subsection reviews the mathematical pipeline that robustly minimizes the obtained *g* function.

### Probability map for stronger edges

#### Probability map

To address the drawback seen at traditional ESFs in edge-based AC, this study introduces a computational pipeline which is based on constructing a robust ESF that utilizes probability scores (between 0−1) rather than predicted class labels provided by a classifier as given in [18]. Probability scores indicate whether a pixel is foreground or background pixel. These scores are computed in *O*(*n*) where n is the number of pixels. Whereas Pratondo et al. [18] uses fuzzy KNN or SVM classifiers to predict whether a pixel is a foreground or background pixel in *O*(*n*^2^). Classifier scores (between 0−1) at boundary pixels tend to be close to zero. So far, considerable amount of work has been done to have ESF collaborate with the likelihood of pixels (whether a pixel belongs to background, foreground, or edge) to avoid contour leakages through the border. Pratondo et al. [18] extended methods of [25, 26] that rely on only class probability using Bayes rule, by utilizing the probability scores from fuzzy KNN and SVM classifiers.

**Fig. 3:**
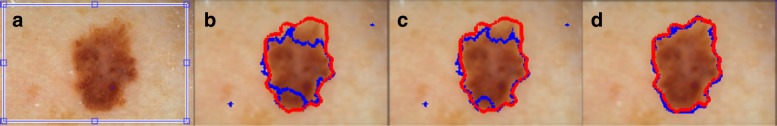
Contribution of attaining a robust edge indicator function is shown. The blue rectangle in **a** marks automatically placed initial contour for each segmentation method. Red line represents the dermatologist drawn ground truth lesion border. Results for Li et al. [28] Pratondo et al. [18], and our method in **b**, **c**, **d** are displayed, respectively, where can be seen that LEEAC outperforms others

We adopted the image segmentation approach studied in [27] that combines pixels ^′^ Gaussian probability distribution (in terms of being a foreground or background pixel) with their geodesic distances to patches selected on foreground and background. Even though this method fails in the dermoscopy images displaying lesion with weak or fuzzy edges, it provides reliable results for mapping probability of pixels that estimates whether they are background or foreground. We approach this feature of [27] such that we minimize the probability matrix where lesion edges are located. Then, we multiply the minimized matrix with the edge indicator function generated by SPH to have a more robust edge indicator function in the segmentation. In our case, object (foreground) is skin lesion and the background is healthy tissue. Figure 3 shows a comparison of segmentation results of methods which use conventional gradients [28], the approach proposed in [18], and our approach to form edge indicator function, respectively.

First step of the probability map generation is to have the regions (boxes) from foreground and background. Boxes (patches) in size of (average) 70x90 collect pixel samples from foreground (lesion) and background (healthy tissue) to create the color models. Pixels on an image will have a value from 0 to 255 at any channel. In probability computation, each of these values is assigned to a probability range between 0 and 1, and the sum of these probabilities for each pixels should be 1. Formal representation is shown as in the Eq. 13,

13 $$  p(I(x,y)=k)  $$

where p represents the probability that the pixel has an intensity of k. Hence, all these can be expressed by a sum as in the Eq. 14.

14 $$  \sum p(I(x,y)=k)=1  $$

**Fig. 4:**
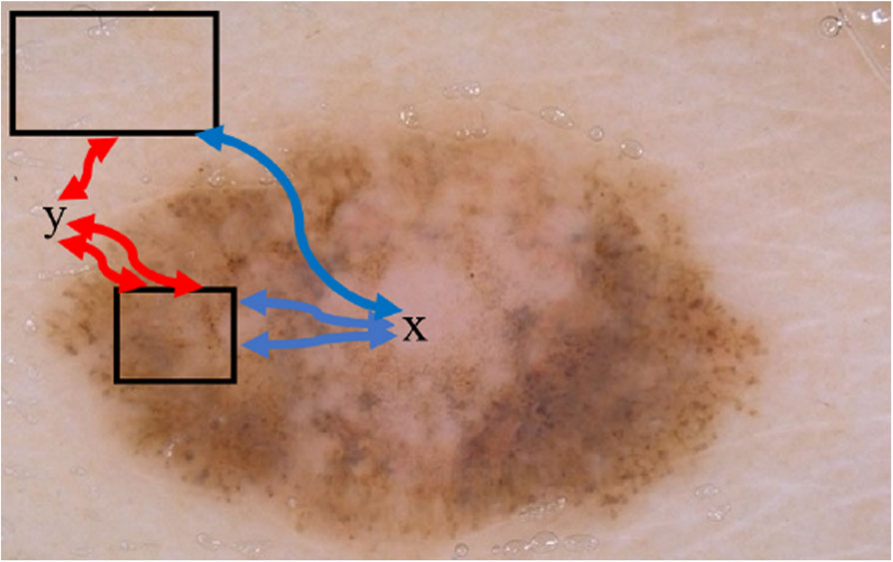
Red and blue curves represent the paths from pixel X to the foreground and background boxes

**Fig. 5:**
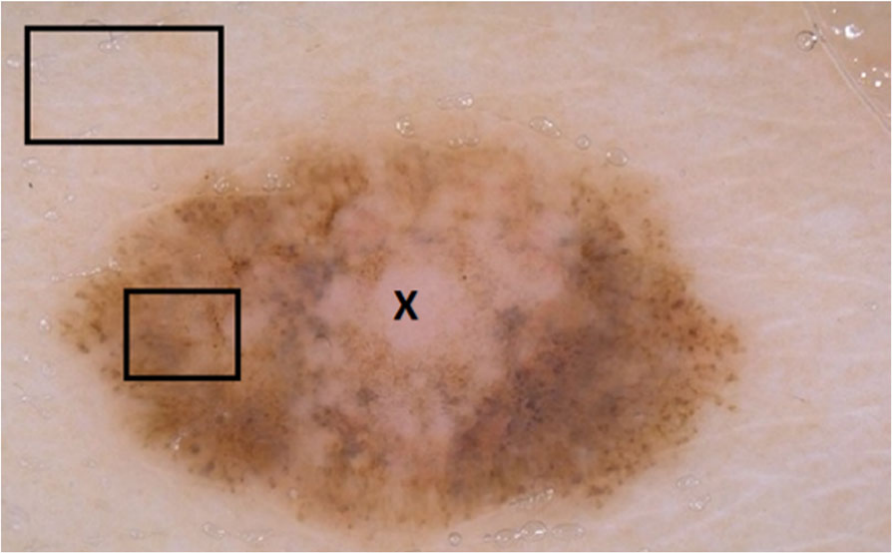
A dermoscopy image displaying a lesion with fuzzy borders and also its interior (as can be seen in pixel X) has similar color features with the background. Border detection for these kinds of lesions is very challenging for most of the methods

To perform background subtraction, let us label the boxes as *l*_1_ and *l*_2_ respectively, where *l*_1_ is from background *Ω*_1_ and *l*_2_ is from foreground *Ω*_2_ of the image *I*. We can approximate the probability density function (PDF) using a Gaussian fitting function shown as in the Eq. 15,

15 $$  p(x)=\frac{1}{\sqrt{2\pi}\sigma}e^{-\frac{{x-\mu}^{2}}{2\sigma^{2}}}  $$

where, *μ* and *σ* represent mean and standard deviation, respectively, estimated on the histogram of data stored in the *l*_1_ and *l*_2_. Figure 4 shows histogram of background and foreground patches obtained from the image displayed in Fig. 5. Using the generic formula given in Eq. 16, the likelihood (in terms of being foreground or background) of a pixel *x* on the channel *C* is given in the Eq. 16.

16 $$  P^{i}_{1|2}(x)= \frac{p^{i}_{1}\left(C_{i}(x)\right)}{p^{i}_{l}\left(C_{i}(x)\right)+p^{i}_{2}\left(C_{i}(x)\right)}  $$

where *p*_*i*_ represents the PDF of *ω*_*j*_ on channel *C*, *i* is the channel number, and in our case *j*=1 and 2 since we have only two labels (foreground and backgorund). Additionally, a weight can be assigned to each channel, then probability of a pixel *x* assigned to *l*_1_ can be computed as in Eq. 17.

17 $$  P^{i}_{1|2}(x)= P_{r}\left(x \in l_{1}\right)=\sum\limits_{i=1}^{N_{c}} w^{i}P^{i}_{1|2}(x)]  $$

where *w*^*i*^ represents the weights which are to impose the channel (*i*∈*N*_*c*_) capacity in terms of abstracting the foreground from background, and *N*_*c*_ represents the channel number.

However, image segmentation merely relies on PDF since probability map is potent to fail. As seen in Fig. 5, pixel *X*, which locates inside of the object of interest, has similar intensity features with the background. In order to address this problem, [27] combined the PDF distribution with geodesic distances of each pixels to these boxes. Following subsection reviews the geodesic distances concept offered by [27].

### Geodesic distances

Geodesic distances are weighted distances. To expand, assume that going to city of B from city of A takes two hours, and distance between A and B is 100 km. Whereas, going to city of C from city of A takes four hours while the distance between A and C is only 50 km. Since C is a city of another country, traveling from A to C requires more effort. Hence, the weight of passing a country border increased the travel time from city A to city C.

Likewise, weighted geodesic distance of each pixels to background and foreground boxes can be computed by Eq. 18 where *W* is the weight, *s*_1_ and *s*_2_ represent the boxes, and *d* represents all possible distances from pixel *X* to background and foreground boxes (see Fig. 4). If the weight *W* is high, then the distance *d* will be high.

18 $$\begin{array}{*{20}l}  d(s)(s_{1},s_{2}) = min\underset{s_{1},s_{2}}{C} \int_{C_{s_{1},s_{2}}}{Wd}_{s} \end{array} $$

Here, pixel assignment to background or foreground is performed by comparing the minimum *distance to the background* and the minimum *distance to the foreground*. Let us select a pixel *X*; if this pixel’s minimum *distance to the background* is less than the minimum *distance to the foreground*, then this pixel is assigned to background, or vice versa.

Geodesic image segmentation selects the gradient of PDF, ∇*P*_*F*,*B*_(*x*), as the weight W. That means, spatial connectivity between observed pixels and pixels of boxes, is constrained by the change of probability (see Fig. 4) as given in Eqs. 19 and 20.

19 $$\begin{array}{*{20}l} W = |\nabla P_{F,B}(x).\overset{\rightarrow}{C^{\prime}}_{s_{1},s_{2}(x)} |  \end{array} $$

20 $$\begin{array}{*{20}l} D_{l}(x) = \underset{s\in\Omega_{1}}{min}\hspace{0.1 cm} d(s,x),l \in\{F,B\}  \end{array} $$

For instance, if ∇*P*_*F*,*B*_(*x*) applies more weight, which means more probability change along the path, in Eq. 19, that yields increase in distance and decrease the possibility of being foreground. Consequently, pixel labeling is conducted by comparing minimum of the distances of *D*_*F*_(*x*) (foreground) and *D*_*B*_(*x*) (background) which are represented in Eq. 20. All of these computations toward generating the probability map are performed in linear time. Interested readers are referred to [27], for more details.

Note that resulting probability maps are used to further strengthen edge indicator function using the formulations given in [18]. Therefore, the edge indicator function will be more robust for the active contour guided image segmentation. Next subsection reviews how we minimize the obtained pixel probability matrix.

#### Minimizing probability matrix

Pixels probability matrix suggests that probability values which are close to 0 and 1 represent background and foreground, respectively; whereas probability values which are close to 0.5 represent edges according to [18]. Pratondo et al. [18] also tries to have more robust edge indicator function to avoid leaks. They applied three different formulas on probability matrix to minimize the values which represent the edges. Finally, they multiplied the minimized probability matrix with their edge indicator function generated from image gradients. In terms of minimizing probability matrix in the edge pixels, we adopt the following formula expressed in Eq. 21 from [18] and replace 0.5 with 0.7 based on our experiments in dermoscopy images.

21 $$  prob(s)=\left.\left(2(s-0.7)^{2}\right)\right)  $$

where s represents the probability matrix for foreground. And new edge indicator function, *g*_*new*_ can be obtained as in the following equation, Eq. 22,

22 $$  g_{new}=g*prob  $$

The equations given in Eqs. 21 and 22 help us minimize the edge indicator function even at the poorly defined object boundaries for the active contours guided segmentation. Therefore, contour evolution will terminate at the desired boundaries.

**Fig. 6:**
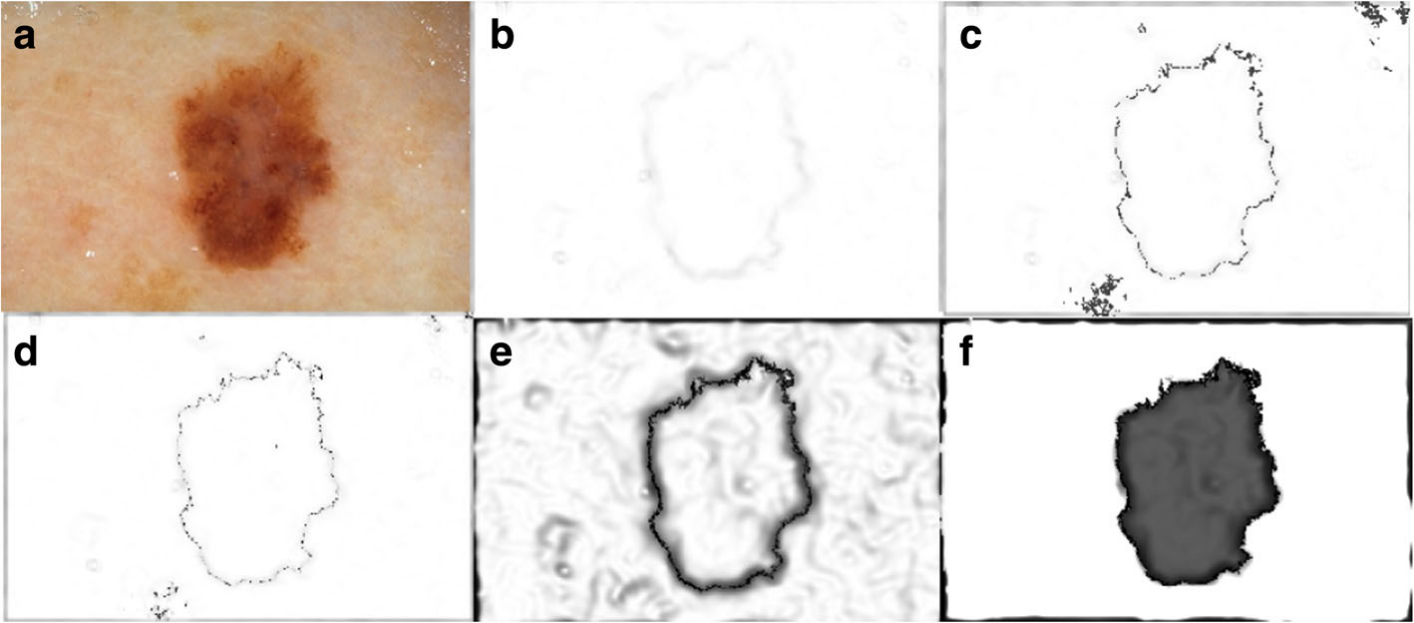
**a** A dermoscopy image with skin lesion, **b** represents a g map obtained using only image gradients, **c** represents a *g* map obtained using SVM and 0.5 in Equation 16, **d** represents a *g* map obtained using KNN and 0.5 in Equation 16, **e** represents a *g* map obtained using geodesic probability map and 0.5 in Equation 16, **f** represents the edge map obtained by geodesic probability map and 0.7 in Equation 16. Additionally we applied a thresholding on **e** to make edges stronger

Figure 6 illustrates the obtained edge map which is a matrix in the image size and will be passed to energy function in the active contours configuration. Note that Fig. 6e and f show more robust edges compared to Fig. 6c and d. Figure 6e and f are created using geodesic probability map. Figure 6b is the conventional edge map that relies solely on image gradients. The enhancement provided by geodesic probability map can be realized qualitatively by naked eye, we also proved it quantitatively using 100 images and displayed the results in the results section. Moreover, training KNN and SVM for the results displayed in Fig. 6c and d are computationally heavy; while in our case, we used geodesic probability map which is obtained in linear time. Average time on generating new edge indicator function for the used data is also discussed in the results section. It is arguable that we continued segmentation after obtaining the outcome shown in Fig. 6e and f. Notably, it is not a binary image that can be used as a segmentation result, and still requires to be processed for abstracting the lesion from background. To address this challenge, we solved the segmentation function (in the spirit of active contours) using level sets. Next subsection reviews the level set evolutions.

## Level set configuration

A level set function (LSF) *ϕ*(*x*,*y*,*t*) can represent a planar closed curve as in the implicit fashion given in Equation 23,

23 $$ CR(t)={(x,y)|\phi(x,y,t)=0}.  $$

where *CR* is curve; *t* is time; and *x* and *y* are spatial coordinates of the given curve. The evolution of the implicit function *ϕ* can be written as in Eq. 24.

24 $$  \frac{\partial \phi}{\partial t} + F\mid\phi\mid = 0  $$

where *F* represents evolution speed and ∇ is gradient operator.

Caselles et al. [14] employed the curve evolution expressed in Eq. 24 for image segmentation. Notion of Caselles’ method relies on constraining curve propagation by image features (commonly gradients). Equation 25 governs the formulations offered by Caselles et al. [14]

25 $$  \frac{\partial \phi}{\partial t} = \mid\phi\mid div\left(g\frac{\nabla \phi}{\mid \nabla \phi \mid}\right)+vg\mid \nabla \phi \mid)  $$

where *g* is the edge indicator function, and *v* represents a constant coefficient that is to be used for adjusting curve speed. Recall that traditional representation of the *g* function is given in Eq. 12. Furthermore, we obtained *g*_*new*_ using geodesic probability map. Consequently, we can now plug our new edge indicator function to level set formulations. Equation 26 is offered by [29] as

26 $$ \begin{aligned} \frac{\partial \phi}{\partial t} = \mu div (d_{p}(\mid \nabla \phi \mid)\nabla \phi) + \\ \lambda \rho(\phi)div\left(g_{new}\frac{\nabla \phi}{\mid \nabla \phi \mid} \right) +\beta g_{new} \rho_{e} (\phi) \end{aligned}  $$

where *d*_*p*_ is obtained from a potential function derived as *d*_*p*_(*s*)=*p*^′^(*s*)/*s*, *δ*_*ε*_ is the Dirac delta function, and *μ*, *λ* and *β* are constants to weight data terms in Eq. 26. The first term on the right side of Eq. 26 is the distance regularization term, the second term represents the length term, and the third term is the area term.

Even though Eq. 26 is able to handle topology changes, that requires re-initialization of level sets, we have adopted a way to tackle re-initialization [30] problem of level set method by using Reaction Diffusion (RD) based Level Set Evolution (LSE) [31]. This method is consisted of two steps such that it first iterates the LSE equation, then solves the diffusion equation. The second step is to regularize the level set function obtained in the first step to remove computationally expensive reinitialization procedure from LSE and to ensure numerical stability in the process of solving Eq. 20. Equation 27 formulizes RD in discrete form as

27 $$ \begin{aligned} \phi^{n+\frac{1}{2}}= \phi^{n} + \tau_{1} \left((\kappa + g_{new}v)+ |\nabla \phi^{n}|\right) \\ \phi^{n+1}=\phi^{n} + \tau_{2}\Delta\phi^{n} \end{aligned}  $$

where, *ϕ* represents the level set function, *ϕ*^*n*^ equals to $\phi ^{n+\frac {1}{2}}$ (for the second raw), *τ*_1_ and *τ*_2_ are time steps of the gradient descent which is to solve the Eq. 27, *κ* is curvature of the level set function, |∇*ϕ*^*n*^| is magnitude of the gradient of the level set function, *Δϕ*^*n*^ is Laplacian of the level set function, *g*_*new*_ is the new edge indicator function, and *v* is a constant to adjust propagation velocity of the level set function. Next section presents the results of our segmentation method including the comparisons with state-of-the art methods.

## Results and discussion

We tested our novel skin lesion border detection method (LEEAC) on the data set that has 100 dermoscopy images provided by [32]. Note that we kept the images in their original sizes to avoid data losses due to down-sampling. We utilized the level set implementation given in [31]. To segment an image, our method requires two patches (boxes); one for background and one for foreground. Then probability map is generated based on the pixels bounded by these rectangular areas. Other algorithms [18, 31, 33] used Gaussian filtering to denoise the data set in their applications, which requires adjusting the standard deviation of Gaussian filter in order to avoid leakages (small values of standard deviation) and long delays in border detection (large values of standard deviation). In this context, delay refers that the initial contour trapped by artifacts in dermoscopy images such as hairs and/or sweat/bubble, ruler markings etc., which cast strong edges on the images.

In SPH map, we used 6^*th*^ polynomial kernel and selected the smoothing range, h as 1. The default parameter values for level set scheme are set as *τ*_1_=0.3, which is the time-step for level set evolution equation; *τ*_2_=0.01, which is the time-step of the equation for diffusion regularization; and *v*=0.7, which is to adjust the speed of curve evolution toward the skin lesion boundary. Iteration loop is stopped when polygon area of the closed contour does not show change more than 10 units compared to area of the contour in previous iteration. In order to conduct a fair comparison, we changed the segmentation configuration given in [18] from [28], to [14], otherwise nested iterations in the implementation of [28] increases computational time drastically. The algorithm of [28] generates inaccurate segmentation results. Note that, in region based segmentation [31, 33] output may contain regions which are not part of the lesion; however, these regions are represented by similar intensity features to the skin lesion. This ultimately decreases their segmentation accuracy. While evaluating our method, we did not take any post-segmentation action such as removing irrelevant connected components (dilation & eroding) far from the lesion to abstract the lesion alone.

To perform evaluation, we adopted commonly used quantitative measurements, i.e., Dice Coefficient (DC), Jaccard Index (JI), and Border Error (BE). Let us say *O* and *G* are the results of segmentation and the ground truth, respectively, DC is calculated by (2∣*O*∩*G*∣)/(∣*O*∣+∣*G*∣), and JI is by (*O*∩*G*)/(*O*∪*G*). BE is calculated as in the Eq. 28.

28 $$ BE=\frac{False Negative+False Positive}{True Negative+True Positive}  $$

**Fig. 7:**
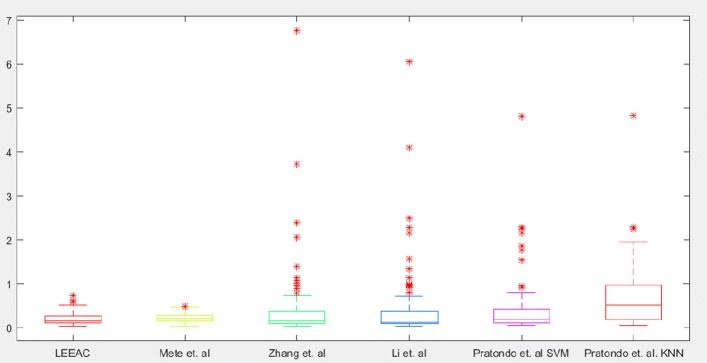
In border error assessment, our method outperformed again with the score of (mean ±standard deviation) 0.1989±0.1428, while Mete et al. [13] reads 0.2273±0.09, Zhang et al. [31] reads 0.406±0.2726, Li et al. [33] reads 0.4061±0.2726, Pratondo [18] reads 0.4079±0.6417 for SVM, and Pratondo [18] reads 0.6276±0.6288 for KNN

**Fig. 8:**
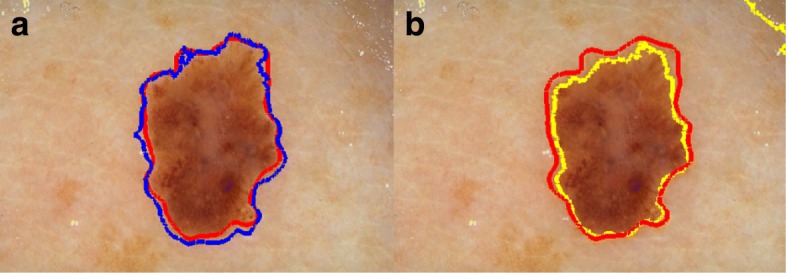
**a** Blue line shows the segmentation result of our method **b** shows the segmentation result of Mete et al. [13] in yellow. Both in (**a**) and (**b**) red line represents the ground truth border of the skin lesion

FN pixels refer to pixels falsely detected as background, FP pixels refer to the pixels falsely segmented as foreground (lesion), TN pixels refer to pixels correctly detected as background, TP pixels correctly segmented as foreground. Figure 7 shows the BE evaluations in the box-plot representation. Table 1 shows comparisons between the prominent segmentation methods [13, 18, 31, 33] and ours. Note that [31, 33] fall in the category of region based active contours, and segmentation functions are governed in the spirit of local binary fitting. Figure 8 includes a gallery that displays qualitative results of our method and competitor methods. Since Pratondo et al. [18] is the only competitor method which is also edge based like ours, our results are especially compared against that method (see Table 1).

**Table 1:** Evaluation of Segmentation Methods with respect to the Ground Truth

Method	Dice	Jaccard	*P*-value
LEEAC	0.8866 ±0.0944	0.8074 ±0.1339	8.16e-14
Zhang et al. [31]	0.8640 ±0.1453	0.7838 ±0.1856	7.33e-11
Li et al. [33]	0.8565 ±0.1681	0.7779 ±0.2021	2.01e-11
Mete et al. [13]	0.8692 ±0.0652	0.7743 ±0.0985	1.08e-14
Pratondo et al. [18] with SVM	0.8395 ±0.1609	0.7511 ±0.1985	3.99e-08
Pratondo et al. [18] with KNN	0.5914 ±0.3564	0.5038 ±0.3392	8.36e-04

**Fig. 9:**
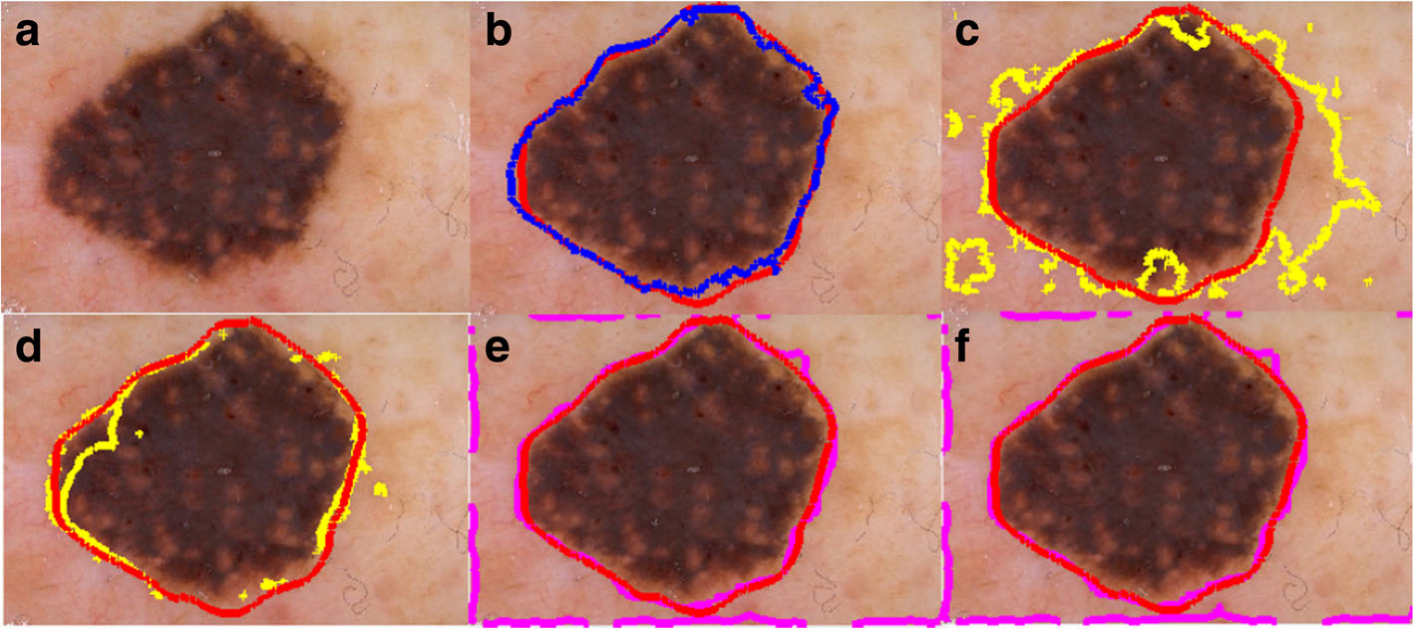
**a** A dermoscopy image of a skin lesion, **b** represents our method (in blue) vs. ground truth (always in red in this figure) lesion border drawn by an expert dermatologist, **c** represents Pratondo et al. [18] using SVM (in yellow) vs. ground truth, and **d** represents Pratondo et al. [18] using KNN (in yellow) vs. ground truth (in blue),**e** represents Zhang et al. [31] (in magenta) vs. ground truth,**f** represents Li et al. [33] vs. ground truth

Mete et al. [13] is a clustering based segmentation method. It is parameter dependent (radius of evolving clusters) and does not incorporate with local information while finding the lesion border. Thus, its segmentation result cannot precisely separate non-lesion patches from lesion if both have similar color values (e.g. fuzzy borders). In Figs. 8 and 9 display qualitative comparisons of our method to competitors [18, 31, 33]. Figure 7 shows the border error evaluations in the box-plot representation.

We have observed that selection of noise filtering technique has a tremendous impact on the duration of segmentation. If we consider images with their actual sizes, methods of [18, 31, 33] used merely Gaussian filtering for denoising purpose and average time for segmenting an image with the size of 484x737 takes more than 10 min. Average time for training KNN and SVM in the approach of [18] is almost an hour. Segmentation method of Mete et al. [13] involves density based clustering, hence it is very sensitive to parameters and computationally expensive.

## Conclusions

This study introduces an accurate skin lesion border detection method based on active contours. One of the main problems of active contours is leaking problem. This problem becomes especially visible in dermoscopy images when there are fuzzy lesion borders and/or dermoscopic artifacts, such as hair and water. When such features exist, active contour is not able to properly find skin lesions or region of interest. We overcome these problems by introducing SPH kernels and probability maps into active contours (called LEEAC). This in turn removed leaking problems and increased accuracy of segmentation. We tested our approach on 100 dermoscopy images and compared our results with the state of the art methods. LEEAC outperforms other prominent methods as reported in the “Results and discussion” section.

## Abbreviations

AC: Active contour
ESF: Edge stop function
FN: False negative
FP: False positive
KNN: K-nearest neighbors algorithm
LEEAC: Local Edge-Enhanced Active Contour
LSE: Level set evolution
LSF: Level set function
PDF: Probability density function
RD: Reaction Diffusion
ROI: Region of interest
SPH: Smoothed particle hydrodynamics
SVM: Support Vector Machines
TN: True negative
TP: True positive
